# Population characteristics, management, and survival outcomes in muscle-invasive urothelial carcinoma undergoing radical resection: the MINOTAUR study

**DOI:** 10.1007/s00345-023-04335-w

**Published:** 2023-03-16

**Authors:** Morgan Roupret, Alice Brouquet, Florian Colrat, Pauline Diez-Andreu, Alexis Prudent, Mélanie Chartier, Anne-Françoise Gaudin, Françoise Bugnard, Louis Chillotti, Stève Bénard, Sébastien Branchoux, Carine Bellera, Sylvie Negrier

**Affiliations:** 1 Sorbonne University, GRC 5 Predictive Onco-Uro, AP-HP, Urology, Pitie-Salpetriere Hospital, Paris, France; 2 Stève Consultants, 30 Rue Narcisse Bertholey, Oullins, France; 3 Bristol Myers Squibb, 3 Rue Joseph Monier, Rueil-Malmaison, France; 4 Inserm CIC1401, Clinical and Epidemiological Research Unit, Institut Bergonié, Comprehensive Cancer Center, INSERM U1219, Bordeaux, France; 5 Lyon I University, Léon Bérard Center, Lyon, France

**Keywords:** Urothelial carcinoma, Insurance claim review, (neo)Adjuvant chemotherapy, Survival outcomes, Bladder cancer, Renal pelvis, Ureter

## Abstract

**Purpose:**

To describe the incidence, management, and survival outcomes of patients with muscle-invasive urothelial carcinoma (MIUC) undergoing radical surgery (RS) in France.

**Methods:**

We relied on a non-interventional real-world retrospective study based on French National Hospitalization Database. Adults with MIUC with a first RS between 2015 and 2020 were selected. Subpopulations of patients with RS performed in 2015 and 2019 (pre-COVID-19) were extracted, according to cancer site: muscle-invasive bladder cancer (MIBC) or upper tract urothelial carcinoma (UTUC). Disease-free and overall survival (DFS, OS – Kaplan–Meier) were assessed on the 2015 subpopulation.

**Results:**

Between 2015 and 2020, 21,295 MIUC patients underwent a first RS. Of them, 68.9% had MIBC, 28.9% UTUC, and 2.2% both cancers. Apart from fewer men among UTUC (70.2%) than MIBC patients (90.1%), patients’ demographic (mean age ~ 73 years) and clinical characteristics were similar whatever the cancer site or year of first RS. In 2019, RS alone was the most frequent treatment, occurring in 72.3% and 92.6% in MIBC and UTUC, respectively. Between 2015 and 2019, neoadjuvant use rate increased from 13.8% to 22.2% in MIBC, and adjuvant use rate increased from 3.7% to 6.3% in UTUC. Finally, median [95% confidence interval] DFS times were 16.0 [14.0–18.0] and 27.0 [23.0–32.0] months among MIBC and UTUC, respectively.

**Conclusion:**

Among patients with resected MIUC annually, RS alone remained the main treatment. Neoadjuvant and adjuvant use increased between 2015 and 2019. Nonetheless, MIUC remains of poor prognosis, highlighting an unmet medical need, notably among patients at high risk of recurrence.

## Introduction

Urothelial carcinomas arise in the bladder (90%) and upper urinary tract (10%). They represent 3% of cancers worldwide and are among the most frequent cancers in France [1]. French Public Health group (*Santé Publique France*) reported in 2018 over 14,000 new cases, with men being mostly affected [1, 2]. Muscle-invasive urothelial carcinomas (MIUCs) encompass both muscle-invasive bladder cancer (MIBC) and upper tract urothelial carcinoma (UTUC). MIUC represents 30% to 50% of urothelial cancers. According to current French and European guidelines, radical surgery (RS)—cystectomy (for MIBC) and/or nephroureterectomy (for UTUC)—is the standard treatment for non-metastatic MIUC. This can be done alone, or with neoadjuvant chemotherapy (NAC) and/or adjuvant chemotherapy (AC) [3–6]. Despite these therapeutics options, MIU﻿Cs remain at risk of recurrence and metastatic evolution given notably their locoregional invasion [7, 8].

Apart from a previous study from 2015 focusing on MIBC patients, there is no recent real-world data on the epidemiology, management, and survival of patients with resectable MIUC in France [5]. The MINOTAUR study therefore aimed to describe the incidence of French patients with MIUC who had undergone RS and their management.

## Methods

### Design

This is a non-interventional, national, real-world, retrospective study using secondary data from the French National Hospitalization Database (PMSI). The PMSI is a hospital claims database containing patient demographic, clinical, and therapeutic data, including diagnosis codes, procedures performed, expensive treatments, and in-hospital deaths for all hospitalizations in public or private hospitals (~ 65 million individuals) [9].

### Study population

Adults with resectable MIUC who underwent a first RS (index date) between January 1, 2015, and December 31, 2020, were included. Patients were identified using validated algorithms integrating diagnoses and procedures according to cancer site (MIBC/UTUC) (see Supplementary material). In case of inconsistency between diagnosis code and performed procedure (e.g., UTUC code with cystectomy), the procedure was favored over diagnosis to determine cancer site. Patients were excluded if they developed metastases within the 3 years before and within 3 months after RS, had neuromuscular dysfunction of the bladder, or any history of urologic surgery before RS.

After exclusion of patients with both MIBC and UTUC at index date, two subpopulations were defined according to the year RS was performed: patients with RS performed in 2015 or in 2019. Patients in the 2015 subpopulation were followed up until December 31, 2020, or until in-hospital death (whichever occurred first), allowing up to 6 years of follow-up for survival analysis. The 2019 subpopulation allowed for detection of potential changes in MIUC management after guideline updates in 2018. The 2020 subpopulation was not used for assessment of changes in management due to the potential effects of the COVID-19 pandemic on healthcare organization and availability, leading to 2019 being the latest and most representative year for management evaluation.

### Outcomes and variables

The primary outcome was the annual incidence of patients that underwent resection of MIUC between 2015 and 2020 according to cancer site (MIBC, UTUC, or both MIBC and UTUC). The secondary outcomes were: patient characteristics, initial treatment of MIUC, in-hospital overall survival (OS), and disease-free survival (DFS), according to cancer site (MIBC or UTUC).

Demographic and clinical characteristics (age, sex, comorbidities, cancer site) were described at index date. Medical history of interest (detailed list available in Supplementary material) and treatment history were assessed for a 3-year period before index date. Initial treatment of MIUC included surgery alone or with NAC, AC, or both. NAC was defined as initiation of chemotherapy within 6 months before RS, and AC as initiation of chemotherapy within 3 months after index date. OS was defined as the time between index date and in-hospital death; no outpatient data are available in the PMSI. DFS was a composite endpoint defined as time from index date to either the date of recurrence or in-hospital death (depending on which event occurred first). Recurrence was defined by a subsequent surgery, lymph node involvement, diagnosis of a new MIUC or metastasis, or initiation of radiotherapy or immunotherapy after index date.

Codes and algorithms for patient selection and definition of variables were derived from validated algorithms [10] and are available in Supplementary material.

### Statistical analyses

Continuous data were reported as means (± standard deviation) and categorical data as counts and proportions. The Kaplan–Meier method was used to estimate survival rates with 95% confidence intervals [CI_95%_] from patients within the 2015 subpopulation with at least 3 months of follow-up after RS. Disease-free and/or surviving patients at last hospital discharge were censored on December 31, 2020. Median follow-up was estimated using the reverse Kaplan–Meier method [11]. Analyses were performed using SAS® version 9.4 (SAS Institute Inc. Cary, NC, USA).

### Ethics

This study was designed according to the International Society for Pharmacoepidemiology (ISPE) guidelines and applicable regulatory requirements, including the French Data Protection Agency (CNIL) act n°2018–257 on regulatory requirements and authorization for processing PMSI data (MR-006) [12–14].

## Results

### Cancer site

**Fig. 1:**
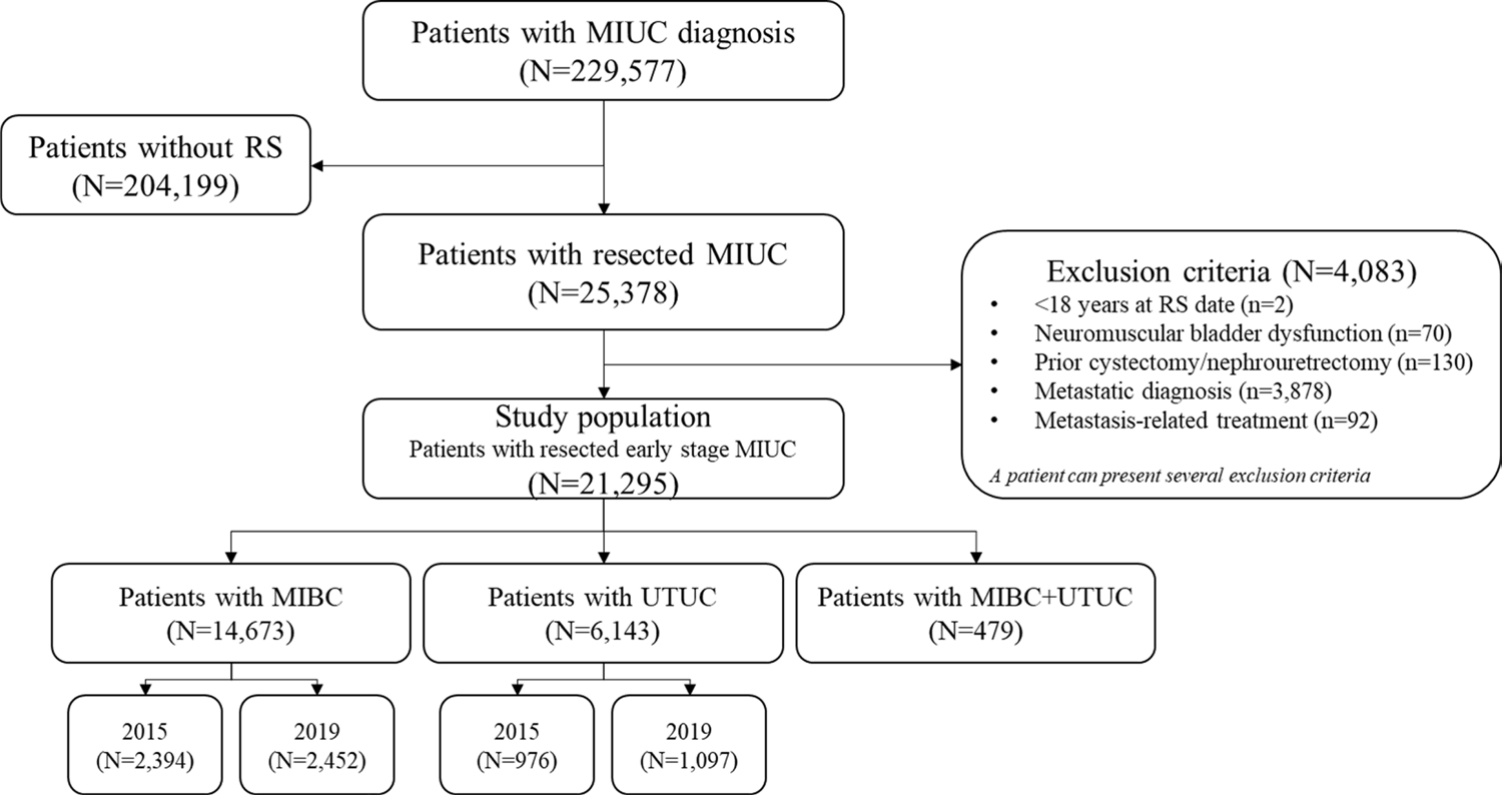
Patient disposition. *MIBC: Muscle Invasive Bladder Cancer; MIUC: Muscle Invasive Urothelial Carcinoma; RS: radical surgery; UTUC: Upper Tract Urothelial Carcinoma*

Between 2015 and 2020, 21,295 patients with MIUC underwent a first RS. Among them, 68.9% (*n* = 14,673) had MIBC, 28.9% (*n* = 6143) had UTUC, and 2.2% (*n* = 479) had both MIBC and UTUC (Fig. 1). During this period, the annual incidence of patients who underwent resection remained stable at approximately 2450 for MIBC and 1,000 for UTUC (Table 1). Median follow-up time was 66.0 months.

**Table 1:** Patients with resected MIUC who underwent a first radical surgery over the 2015–2020 period

	MIBC patients (*N* = 14,673)	UTUC patients (*N* = 6143)	Both MIBC and UTUC (*N* = 479)	Overall (*N* = 21,295)
2015	2,394	976	109	3,479
2016	2,393	962	89	3,444
2017	2,489	1,028	96	3,613
2018	2,470	1,047	80	3,597
2019	2,452	1,097	68	3,617
2020	2,475	1,033	37	3,545

*MIBC: Muscle Invasive Bladder Cancer; UTUC: Upper Tract Urothelial Cancer*

### MIBC

In 2015, 2,394 patients with MIBC underwent a first RS. The mean age was 72.1 (± 9.5) years, with 78.0% of patients aged ≥ 65 years, and 90.1% of patients were male (Table 2). The most frequent comorbidities were diabetes (*n* = 524, 21.9%), obesity (*n* = 397, 16.6%), kidney disease (*n* = 381, 15.9%), chronic pulmonary disease (*n* = 349, 14.6%), peripheral vascular disease (*n* = 287, 12.0%), and myocardial infarction (*n* = 239, 10.0%). The sociodemographic and clinical characteristics were similar between the 2015 and 2019 subpopulations (Table 2).

**Table 2:** Sociodemographic and clinical characteristics of patients with resected MIBC or UTUC in 2015 and 2019

	MIBC		UTUC	
	2015 (*n* = 2394)	2019 (*n* = 2452)	2015 (*n* = 976)	2019 (*n* = 1097)
Gender				
Female *n* (%)	237 (9.9%)	212 (8.6%)	291 (29.8%)	351 (32.0%)
Age				
Mean (SD)	72.1 (9.5)	72.5 (9.2)	71.6 (10.5)	71.7 (9.5)
Median (Q1-Q3)	73.0 (66.0–79.0)	73.0 (67.0–79.0)	73.0 (65.0–79.0)	72.0 (66.0–78.0)
≥ 65y *n* (%)	1,867 (78.0%)	1,982 (80.8%)	746 (76.4%)	868 (79.1%)
Comorbidities of interest *n* (%)				
Diabetes	524 (21.9%)	544 (22.2%)	173 (17.7%)	222 (20.2%)
Obesity	397 (16.6%)	424 (17.3%)	136 (13.9%)	164 (14.9%)
Kidney disease	381 (15.9%)	420 (17.1%)	153 (15.7%)	193 (17.6%)
Chronic pulmonary disease	349 (14.6%)	323 (13.2%)	86 (8.8%)	117 (10.7%)
Peripheral vascular disease	287 (12.0%)	288 (11.7%)	87 (8.9%)	98 (8.9%)
Myocardial infarction	239 (10.0%)	224 (9.1%)	70 (7.2%)	74 (6.7%)
Congestive heart failure	158 (6.6%)	145 (5.9%)	42 (4.3%)	42 (3.8%)
Cerebrovascular disease	144 (6.0%)	125 (5.1%)	50 (5.1%)	50 (4.6%)
Hemiplegia	62 (2.6%)	57 (2.3%)	9 (0.9%)	13 (1.2%)
Mild liver disease	39 (1.6%)	45 (1.8%)	15 (1.5%)	14 (1.3%)
Dementia	34 (1.4%)	38 (1.5%)	4 (0.4%)	10 (0.9%)
Ulcer disease	30 (1.3%)	42 (1.7%)	9 (0.9%)	14 (1.3%)
Rheumatic disease	17 (0.7%)	24 (1.0%)	2 (0.2%)	11 (1.0%)
Severe liver disease	13 (0.5%)	18 (0.7%)	5 (0.5%)	4 (0.4%)
AIDS/HIV	4 (0.2%)	5 (0.2%)	2 (0.2%)	0 (0.0%)
Treatment type *n* (%)				
Surgery alone	1,932 (80.7%)	1,773 (72.3%)	928 (95.1%)	1,016 (92.6%)
NAC treatment	330 (13.8%)	544 (22.2%)	10 (1.0%)	10 (0.9%)
AC treatment	119 (5.0%)	112 (4.6%)	36 (3.7%)	69 (6.3%)
Both NAC and AC	13 (0.5%)	23 (0.9%)	2 (0.2%)	2 (0.2%)

*AC: Adjuvant Chemotherapy; AIDS/HIV: Acquired Immune Deficiency Syndrome/Human Immunodeficiency Virus; MIBC: Muscle Invasive Bladder Cancer; NAC: Neoadjuvant Chemotherapy; UTUC: Upper Tract Urothelial Cancer; RS: radical surgery*

RS alone was the most common treatment for patients with MIBC in 2015 (*n* = 1932, 80.7%). NAC utilization increased between 2015 and 2019 for patients with MIBC (13.8%, *n* = 330 for 2015 and 22.2%, *n* = 544 for 2019). Both AC and NAC/AC utilization remained stable between 2015 and 2019 (~ 5% for AC and ~ 0.5% for NAC/AC).

**Fig. 2:**
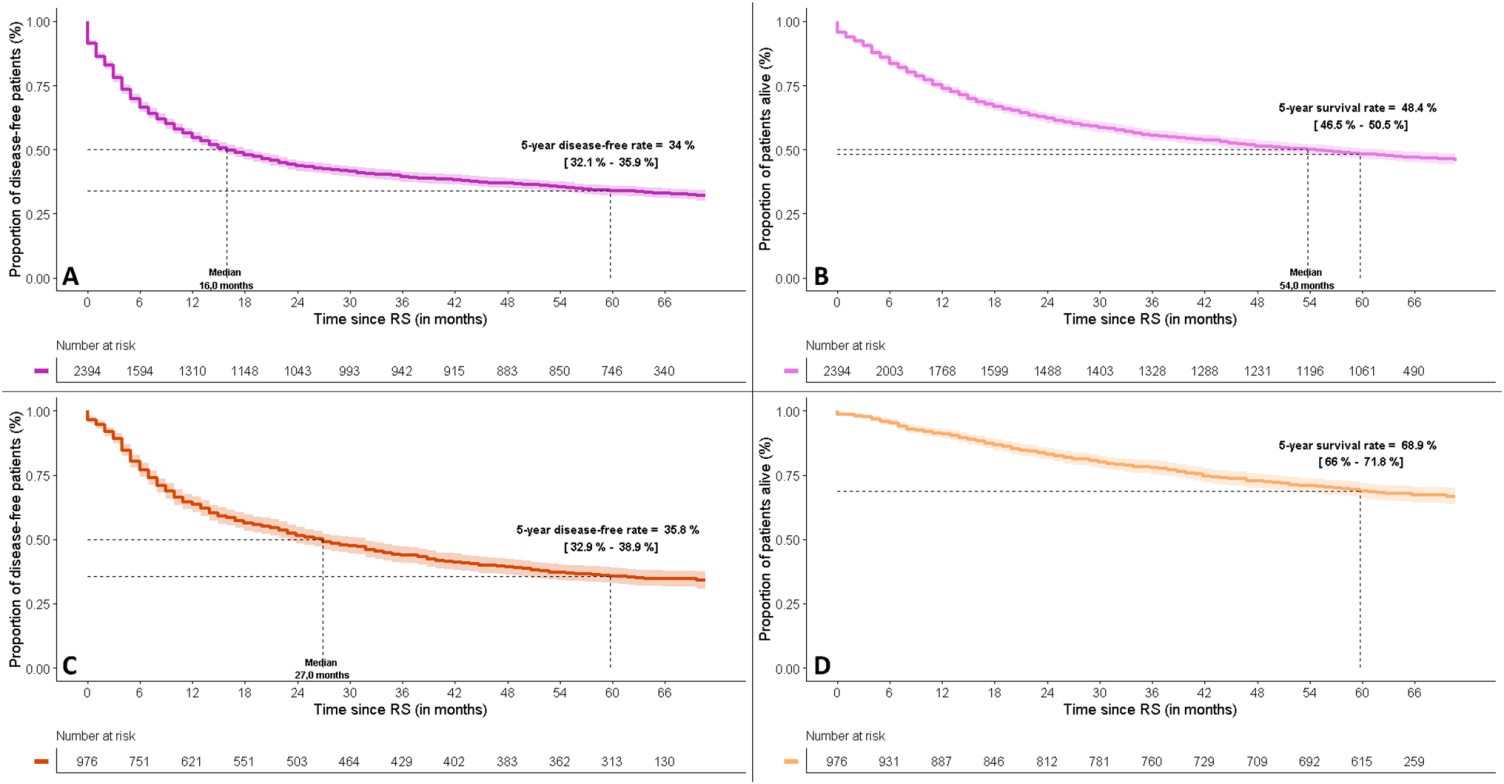
DFS and OS after RS in patients with resected MIBC and UTUC—Kaplan–Meier method. *DFS: Disease-free survival; MIBC: Muscle Invasive Bladder Carcinoma; OS: Overall Survival; RS: radical surgery; UTUC: Upper Tract Urothelial Carcinoma.* (A) MIBC DFS, (B) MIBC OS, (C) UTUC DFS, (D) UTUC OS

The 24-month DFS rate [CI_95%_] was 43.6% [41.6–45.5], and 24-month OS rate was 62.2% [60.2–64.1]. The 60-month DFS rate [CI_95%_] was 34.0% [32.1–35.9], and 60-month OS rate was 48.4% [46.5–50.5] (Fig. 2A, B). Median DFS and OS times were 16.0 months [14.0–18.0] and 54.0 months [47.0–63.0], respectively.

### UTUC

In 2015, 976 patients with UTUC underwent a first RS. The demographic and clinical characteristics were similar to those of patients undergoing resection of MIBC except for a slightly lower proportion of males (70.2%: Table 2).

RS alone was the most frequent treatment for patients with UTUC in both 2015 (*n* = 928, 95.1%) and 2019 (*n* = 1016, 92.6%). AC utilization increased between 2015 and 2019 for patients with UTUC (3.7% (*n* = 36) in 2015 and 6.3% in 2019 (*n* = 69)). NAC (~ 1%) and both NAC/AC (~ 0.2%) utilization remained very low and stable between 2015 and 2019.

The 24-month DFS rate [CI_95%_] was 51.5% [48.4–54.6], and 24-month OS rate was 83.2% [80.7–85.4]. The 60-month DFS rate [CI_95%_] was 35.8% [32.9–38.9], and 60-month OS rate was 68.9% [66.0– 71.8] (Fig. 2C, D). Median DFS time was 27.0 months [23.0–32.0]. Median OS time was not reached.

## Discussion

This is the first study providing real-world data on the peri-operative management and the survival outcomes of MIUC patients treated by RS in France.

Sociodemographic characteristics were consistent with prior literature. The median age was 73 years and most of patients were male (approximately 75%) [2, 15, 16]. Chronic pulmonary disease and obesity are two known risk factors for urothelial cancers. Both factors were the most frequent comorbidities and concerned more than 12% of patients included. This percentage is likely underestimated, as for other comorbidities, due to PMSI data availability limitations [17–19].

Among patients who underwent surgery, 30% were UTUC patients. This is a higher rate than currently described in the literature indicating approximately 10% [1, 7]. This could be explained by our patient selection criteria. Indeed, we excluded patients with no indication of surgery (notably those with metastatic MIUC at time of diagnosis) as well as those with a history of partial surgery for MIUC. This could have led to a higher proportion of patients with UTUC, since patients with this cancer site are more likely to be indicated for surgery based on the staging, compared to patients with MIBC [20].

The current French and European guidelines consider RS, with cisplatin-based NAC or AC for eligible patients, as the standard treatment for the management of resectable MIUC [3–6]. This study highlighted that RS alone was the most frequent initial management for resectable MIUC. Independent of cancer site or year of surgery, over 70% of patients with MIUC underwent RS alone. This incidence even reached 92% of patients with UTUC in 2019.

However, an increase in NAC use was observed among patients with MIBC, reaching 22.2% in 2019, lower than the 50% proportion of patients expected to be fit for it (maximum) [21]. A similar but smaller increase was observed for AC use in patients with UTUC, reaching 6% in 2019. This could depict the progressive implementation in routine practice of successive guidelines [22, 23]. However, it cannot be ruled out that a change in epidemiology of MIUC (proportion of patients with MIBC/UTUC, severity, resectability) could underlie this change [6].

A median DFS time of 16.0 months was reported for patients with MIBC and 27.0 months for patients with UTUC after resection. These results are consistent with international reports. After resection, Drakaki et al. and Birtle et al. demonstrated a median DFS of 13.5 and 29.8 months for patients with MIBC or UTUC, respectively [24, 25]. The 5-year survival rate after resection for MIBC is < 50% according to the French health authorities and the French National Cancer Institute, which is also in line with current findings [26].

This study used the French PMSI, therefore ensuring exhaustive data analysis and accurately reflecting the current epidemiology and management of MIUC in France. The study design combined medical history assessment up to 3 years before index date and up to 6 years of follow-up for the 2015 subpopulation, allowing for a thorough analysis of patients with MIUC after the first RS as well as assessment of DFS and OS.

On top of being retrospective, the main limitation related to PMSI is the lack of clinical data (e.g., TNM stage, histological results). This limitation has been compensated for by the development of algorithms and medical reviews, as confirmed by our results which are consistent with the literature [11, 23]. However, since risk assessment or documentation of previous history of non-MIBC (based on staging) of included patients was not possible, the study included heterogeneous patients with both low and high risk of recurrence or death, and patients with either primary or secondary MIBC [28]. These latter could have lower survival outcomes [29].

Another limitation is the absence of outpatient data, meaning that OS and DFS rates were estimated considering in-hospital death only, which could have led to their underestimation for the overall French MIUC population. However, results were consistent with the literature. This implies a low rate of outpatient mortality, as already highlighted by a study from *Santé Publique France* showing that 70–80% of patients with cancer die in hospital [30].

Overall, the French PMSI is a robust and exhaustive data source for cancer epidemiology, management, and survival [9]. Despite not describing the effects of using new drugs at different disease stages (e.g., adjuvant, metastatic), our findings are representative of the current management strategies in place for MIUC in France.

## Conclusions

This study shows that RS alone remains the main treatment for MIUC initial management. Minor changes in practices were observed between 2015 and 2019, with an increase in NAC use among patients with MIBC and a slighter increase in AC use among patients with UTUC.

Survival among patients undergoing MIUC resection remained poor with a median DFS time of approximately 2 years and a median OS time shorter than 5 years among patients with MIBC. This highlights an unmet medical need which could be partly tackled by the emergence of innovative treatments.

## Declarations

### Data availability statement

The source data base (PMSI) contains personal health data containing potentially identifying and sensitive patient information. In accordance with CNIL opinion n°2018-257 of 7th June 2018 on regulatory requirements and governance for processing PMSI data under MR-006 procedure, no raw data can be shared externally. The request to access PMSI data must be submitted to the Health Data Hub, the French public agency which gathers the requests, as per the MR-006 procedure. We are not permitted to publicly share these data.
